# Ultra-Strong Transparent ZnAl_2_O_4_ Glass-Ceramics via Controlled Crystallization and Ion Exchange

**DOI:** 10.3390/ma18225230

**Published:** 2025-11-19

**Authors:** Ivan Veselov, Georgiy Shakhgildyan, Vitaliy Savinkov, Nikita Golubev, Kirill Tregubov, Daniil Vinogradov, Leon Avakyan, Michael Ojovan, Manasi Ghosh, Vladimir Sigaev

**Affiliations:** 1 Department of Glass and Glass-Ceramics, Mendeleev University of Chemical Technology, Moscow 125047, Russia; ivan.veselov.115@gmail.com (I.V.); sav.vi@mail.ru (V.S.); golubev_muctr@mail.ru (N.G.); kirill.tregubov.2018@gmail.com (K.T.); vdvhostagedaniil@yandex.ru (D.V.); vlad.sigaev@gmail.com (V.S.); 2 Physics Faculty, Southern Federal University, Rostov-on-Don 344090, Russia; laavakyan@sfedu.ru; 3 School of Chemical, Materials and Biological Engineering, The University of Sheffield, Sheffield S1 3JD, UK; m.ojovan@sheffield.ac.uk; 4 Physics Section, MMV, Banaras Hindu University, Varanasi 221005, India; manasi.ghosh@bhu.ac.in

**Keywords:** transparent glass-ceramics, glass crystallization, gahnite, ion exchange, Vickers hardness, glass strengthening

## Abstract

Enhancing the mechanical strength of transparent glass-ceramics (TGCs) without compromising their optical performance remains a key challenge for advanced optical and photonic materials. Among aluminosilicate systems, ZnO–MgO–Al_2_O_3_–SiO_2_ (ZMAS) glasses are particularly attractive due to their ability to form ZnAl_2_O_4_-based nanostructures; however, their ion-exchange (IE) strengthening has not been systematically explored due to the absence of single-charged cations in their composition. In this study, a sodium-modified ZMAS glass was developed to enable efficient chemical strengthening while preserving glass-forming ability and optical clarity. Controlled two-stage heat treatment produced TGCs containing 5 mol% Na_2_O, composed solely of ZnAl_2_O_4_ (gahnite) nanocrystals with an average size of 4–5 nm. The obtained TGCs showed a Vickers hardness of ~8.5 GPa, increasing to ~10–10.5 GPa after ion exchange in molten KNO_3_ at 450 °C, without changes in phase composition or optical transmittance. Compared with literature data on alkali-containing TGCs, the developed material demonstrates a higher hardness level while maintaining full transparency. The results reveal a practical route toward chemically strengthened ZnAl_2_O_4_-based glass-ceramics combining optical clarity, high hardness, and damage tolerance for optical, photonic, and protective applications.

## 1. Introduction

Improving the mechanical reliability of transparent glasses an d glass-ceramics remains a key challenge for applications in aerospace systems, optoelectronics, precision optics, and photonic devices, where high optical clarity must be combined with resistance to surface damage and cracking. Two industrially mature routes are widely used to increase strength: thermal tempering [1] and chemical strengthening by ion exchange (IE) [2,3,4]. In parallel, the transition from a homogeneous glass to a glass-ceramic, via controlled crystallization, often provides a substantial gain in hardness and damage tolerance while preserving transparency when the microstructure is nanometric and uniformly distributed [5,6,7,8].

Ion-exchange strengthening of transparent glass-ceramics (TGCs) is particularly attractive because a compressive surface layer can be superimposed on a fine, mechanically robust glass-ceramic framework. Successful IE of glass-ceramics, however, requires that the residual glassy phase contains a sufficient concentration of alkali cations amenable to substitution (Li^+^/Na^+^ → K^+^). Compositional tuning to increase the alkali content may, in turn, alter the crystallization pathway—phase selection, kinetics, and even the balance between bulk and surface crystallization—and thereby jeopardize transparency if coarse or mismatched phases precipitate.

Among aluminosilicate families, the most explored chemistries for TGCs strengthened by IE include Li_2_O–Al_2_O_3_–SiO_2_ (LAS) [9,10,11,12], MgO–Al_2_O_3_–SiO_2_ (MAS) [13], ZnO–Al_2_O_3_–SiO_2_ (ZAS) [14,15,16,17], and their combinations (e.g., ZMAS) [13,18,19]. To enhance IE efficiency, Na_2_O is often introduced into LAS-type glasses to facilitate Li^+^→Na^+^→K^+^ exchange, either in one step (KNO_3_/NaNO_3_ melts) or in staged protocols (Na^+^ exchange followed by K^+^ exchange) [10,15]. Table 1 compiles representative literature data for TGCs containing alkali species, covering salt compositions, IE schedules, and the resulting mechanical properties. Despite differences in test conditions (especially Vickers loads), these reports generally place pre-IE Vickers hardness in the range ≈6–8 GPa; post-IE strengthening typically raises microhardness to ≈7–9 GPa.

Within ZMAS-type systems, IE-compatible formulations pose a particular challenge. For example, in a Na_2_O-rich composition (6ZnO–12MgO–8Na_2_O–17Al_2_O_3_–52.5SiO_2_–3TiO_2_–1.5ZrO_2_) [13], part of Na_2_O is consumed by Na_2_TiSiO_5_ crystallization alongside spinels (ZnAl_2_O_4_/MgAl_2_O_4_); the highest reported hardness (~8.2 GPa) was accompanied by a transmittance of only ~60–80% (380–780 nm) for 2 mm thick samples, likely due to coarse (>40 nm) crystals. In contrast, low-Na ZMAS glass (1.1 mol% Na_2_O) crystallized into a ZnAl_2_O_4_-based TGC with microhardness near 9 GPa [23]; however, the low alkali content limits the subsequent IE efficiency. Recent work demonstrated that increasing Na_2_O to 5 mol% preserves glass-forming ability (bulk casts ~200 g without devitrification) and enables moderate strengthening of the glass by IE (from 6.7 to 8.1 GPa) [24]. The crystallization behavior of this 5 mol% Na_2_O ZMAS composition, its ability to yield a transparent spinel-based TGC, and its response to ion exchange have not yet been established.

Despite extensive studies on lithium- and magnesium-aluminosilicate systems, the development of chemically strengthened transparent glass-ceramics in the ZnO–MgO–Al_2_O_3_–SiO_2_ family remains limited. The main challenge lies in achieving an alkali-rich residual glass phase—necessary for efficient ion exchange—while maintaining controlled bulk crystallization that preserves optical transparency.

In this context, the present study explores a Na_2_O-modified ZMAS glass designed to balance glass-forming ability, nanocrystallization behavior, and ion-exchange capability. We show that moderate Na_2_O incorporation enables the formation of a fully transparent ZnAl_2_O_4_-based nanostructure suitable for subsequent ion-exchange strengthening without altering the phase composition or optical clarity. This approach advances the understanding of how nanostructural design and ion-exchange strengthening can be effectively combined in ZMAS-type glass-ceramics, opening opportunities for the development of mechanically durable transparent materials for optical, photonic, and protective applications.

## 2. Materials and Methods

### 2.1. Glass Composition and Batching

The target (nominal) glass composition investigated in this work (hereafter 5Na, mol%) was: 20.1 ZnO; 10.0 MgO; 5.0 Na_2_O; 9.8 Al_2_O_3_; 48.0 SiO_2_; 5.9 TiO_2_; 1.2 ZrO_2_. This composition features a ZnO/MgO ratio of ~2:1 and thus differs markedly from the formulation reported in [13], where ZnO/MgO ≈ 1:2. Analytical-grade raw materials were used: SiO_2_ (≥99.99%), ZrO_2_ (≥99.99%), ZnO (≥99.5%), TiO_2_ (≥99.99%), Na_2_CO_3_ (≥99.8%), MgO (≥98.0%), and Al(OH)_3_ (≥97.0%). The weighed powders were homogenized by mechanical mixing for 4 h and then charged into a corundum crucible.

### 2.2. Melting, Casting, and Annealing

Melting was carried out in a bottom-loading furnace equipped with MoSi_2_ heating elements. The batch was loaded into a corundum crucible (Al_2_O_3_, 99.9%) and heated to 1550 °C, held for 1 h at the maximum temperature, and the melt was then cast into a preheated metal mold to produce bulk glass. The castings were annealed at 550 °C for 4 h in a muffle furnace to relieve residual internal stresses.

### 2.3. Controlled Crystallization

Annealed glasses were cut and polished, then subjected to a two-step heat treatment in a muffle furnace. The nucleation stage was performed at 660 °C for 4 h (in the vicinity of Tg), followed by a crystal-growth stage conducted at temperatures near the first DSC exotherm for 10–110 h. The resulting TGC are denoted 5Na–X–Y, where X is the heat treatment temperature and Y is the holding time (h).

### 2.4. Ion Exchange Treatment

Ion exchange (IE) of both the parent glass and the TGC was conducted in a pure KNO_3_ melt at 450 °C for 12, 24, 48, 72, 96, and 120 h. After IE, samples were withdrawn from the salt bath and subjected to inertial cooling in a preheated muffle furnace to minimize thermal shock and relax thermal stresses. Residual salts were removed by rinsing in distilled water.

### 2.5. Characterization of Structure and Properties

Differential scanning calorimetry (DSC) was performed by means of a thermal analyzer STA 449 F3 Jupiter (Netzsch, Selb, Germany) in a Pt crucible, at a heating rate of 10 °C/min in Ar. The glass transition temperature (Tg) was determined as the extrapolated onset of the transition, while exopeak temperatures (Tp) were defined as the peak extremum temperature in DSC curves. X-ray diffraction (XRD) patterns of powdered samples were recorded by means of a diffractometer D2 Phaser (Bruker, Karlsruhe, Germany) employing nickel-filtered CuKα radiation. Crystalline phases were identified by comparing the peak position and relative intensities in the X-ray diffraction pattern with the ICDD PDF-2 database. XRD data were collected between 10 and 60 2θ, with a step size of 0.02° and a counting time of 0.3 s per step. XRD patterns were refined using the Rietveld method as implemented in the GSAS-II v5.6.0 code [25]. Optical transmission spectra were recorded over 300–800 nm using a UV-Vis spectrophotometer (UV-3600, Shimadzu, Kyoto, Japan) on the 1.5 mm thick samples. The microstructure of the glass and TGC samples was examined using high-resolution transmission electron microscopy (HRTEM, JEM-2100Plus, JEOL, Peabody, MA, USA) operated at an accelerating voltage of 200 kV. Bulk glass samples were ground to fine powders in an agate mortar and ultrasonically dispersed in ethanol. A drop of the resulting suspension was deposited onto a carbon-coated copper grid and dried under ambient conditions for approximately 20 min. Bright-field TEM images were recorded from multiple regions of each grid to obtain statistically representative microstructural data. The digital micrographs were analyzed using ImageJ software (version 1.53n). NTEGRA Spectra Spectrometer (NT-MDT Co., Zelenograd, Moscow, Russia) with the Ar laser beam (488 nm excitation wavelength) was used to record Raman spectra of the bulk samples.

Vickers microhardness was measured using an HVS-1000 tester (Laizhou Huayin Testing Instrument Co., Ltd., Laizhou, Shandong, China) under a 200 g load with a 10 s dwell time. The Vickers hardness was calculated as

(1) $$H_{V}=1.854\;\frac{F}{d^{2}}\;$$

where *F* is the applied load and *d* is the mean indentation diagonal. For each specimen, ≥20 indents were performed for statistical reliability.

## 3. Results and Discussion

### 3.1. Structure and Thermal Behavior of the Parent Glass

The glass composition containing 5 mol% Na_2_O was selected based on our previous research [24]. An increase in Na_2_O content beyond this level led to partial crystallization during casting, hindering the formation of a homogeneous bulk glass. To determine the characteristic thermal transitions, DSC was performed on monolithic samples of the 5Na glass.

**Figure 1:**
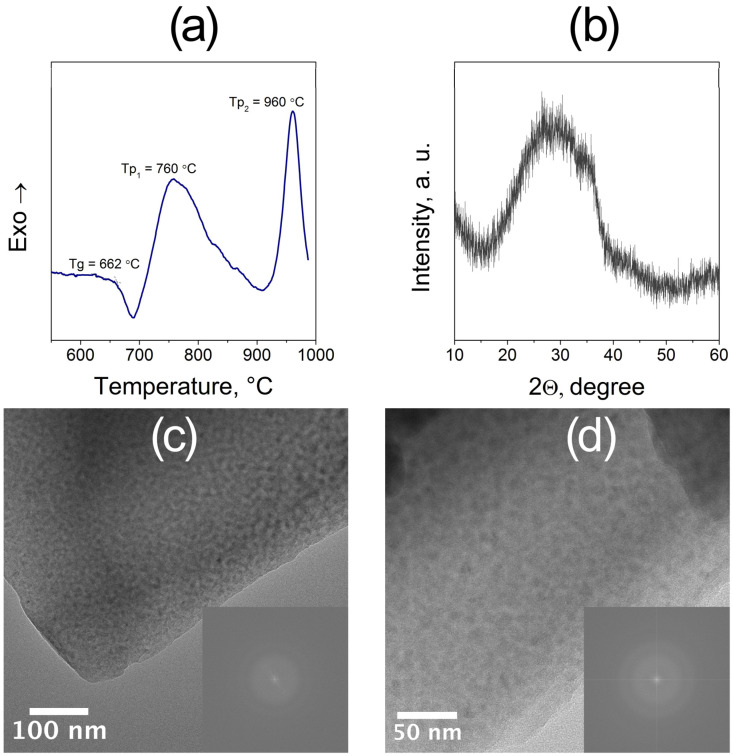
(**a**) DSC curve of the parent glass showing the glass transition temperature and two exothermic peaks corresponding to the sequential crystallization stages. (**b**) XRD pattern of parent glass confirming its amorphous structure. (**c**,**d**) HRTEM images of parent glass at different magnifications with corresponding SAED patterns (insets) showing diffuse rings typical of an amorphous matrix.

As shown in Figure 1a, the DSC curve exhibits a distinct glass transition at 662 °C, followed by two exothermic peaks at 760 °C (Tp_1_) and 960 °C (Tp_2_), corresponding to the primary and secondary crystallization stages, respectively. These data were used to establish the temperature parameters for controlled nucleation and crystal growth in the subsequent heat-treatment procedure.

To evaluate the relationship between surface and bulk crystallization, DSC measurements were also conducted on glass samples of identical mass in both powdered and monolithic forms (Figure S1). The two DSC curves display nearly identical shapes, with only a slight shift of the exothermic peaks toward higher temperatures for the powdered sample. This minimal difference indicates that the crystallization of the 5Na glass proceeds predominantly via a bulk mechanism. This conclusion is further supported by the complete coincidence of the XRD patterns obtained for powdered and monolithic samples heat-treated under identical conditions (660 °C—4 h—760 °C—10 h, Figure S2).

The amorphous structure of the 5Na glass was confirmed by XRD and TEM analyses. The XRD pattern exhibits a single broad diffuse halo centered near 2θ ≈ 30° (Figure 1b), confirming the absence of crystalline phases. TEM images of the glass show overall homogeneous contrast, yet reveal faint nanoscale compositional modulations, indicative of liquid–liquid phase separation typical for ZMAS-type glasses (Figure 1c,d). The corresponding SAED patterns display diffuse rings without discrete reflections, confirming that both phases remain fully amorphous. Such phase separation is a well-known precursor for controlled crystallization in ZnO/MgO–Al_2_O_3_–SiO_2_ glasses, providing chemically enriched regions that subsequently act as favorable sites for nucleation of crystallites during heat treatment.

The relatively high glass transition temperature (Tg = 662 °C) of the 5Na composition, well above the typical ion-exchange temperature (≈450 °C), implies that structural relaxation during ion exchange is negligible, ensuring the stability of the glass matrix during chemical strengthening. Structural relaxation refers to the temperature-dependent rearrangement of the glass network toward equilibrium through viscous flow or atomic diffusion; at temperatures far below Tg, the glass structure remains rigid and cannot relax the stresses generated during ion exchange.

### 3.2. Crystallization Behavior and Phase Evolution

The temperature–time parameters for two-step crystallization were selected following the approach previously applied to transparent ZMAS glass-ceramics with 1.1 mol% Na_2_O [23]. The nucleation stage was carried out at 660 °C for 4 h (near Tg), followed by a crystal growth stage within 750–850 °C for 10–110 h. Samples heat-treated at 750–760 °C for 10 h retained full transparency, while increasing the temperature to 770 °C led to slight opalescence, and further heating to 785 °C and above produced opaque glass-ceramics.

**Figure 2:**
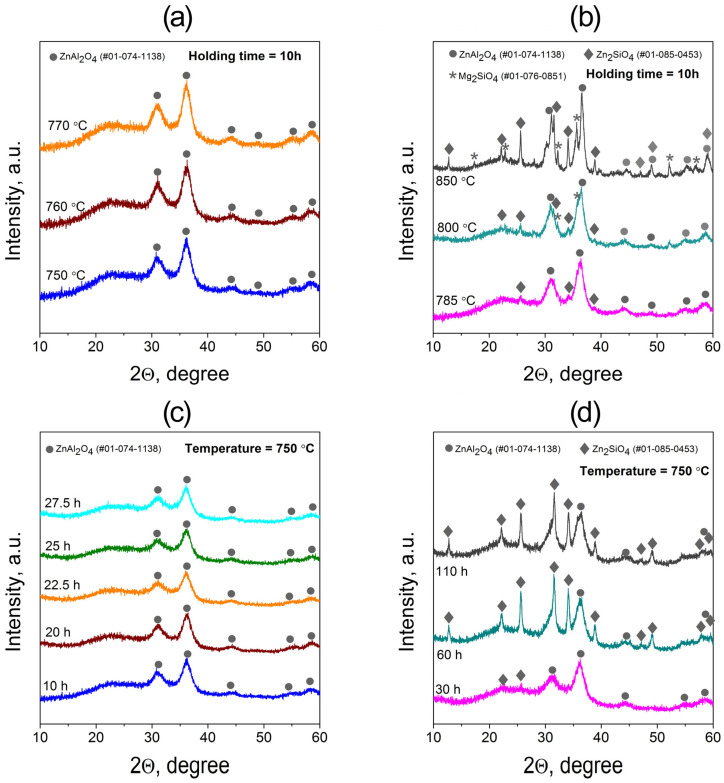
XRD patterns of the 5Na TGC heat-treated at different temperatures and durations: (**a**,**c**) transparent samples containing only ZnAl_2_O_4_ spinel; (**b**,**d**) opaque samples containing additional Zn_2_SiO_4_ and Mg_2_SiO_4_ phases.

The evolution of crystalline phases depending on temperature and holding time is shown in Figure 2. At 750–770 °C (Figure 2a), all diffraction peaks correspond to the zinc aluminate ZnAl_2_O_4_ (PDF #01-074-1138), indicating that a single spinel-type phase is formed in this temperature range. Further heating to 785–800 °C (Figure 2b) results in the appearance of additional reflections assigned to zinc silicate Zn_2_SiO_4_ (PDF #01-085-0453). The intensity of these peaks increases with temperature, and at 850 °C, weak peaks of forsterite Mg_2_SiO_4_ (PDF #01-076-0851) also appear. At this stage, silicate phases dominate over the spinel, causing a pronounced decrease in transparency.

For samples treated isothermally at 750 °C for 10–110 h (Figure 2c,d), the diffraction data reveal a time-dependent phase evolution. Up to ~25 h, ZnAl_2_O_4_ remains the only crystalline phase, while its mean crystallite size, calculated using the Scherrer equation from the (311) reflection at 36.3°, remains constant at ~4–5 nm. Prolonged holding (>60 h) promotes the formation of Zn_2_SiO_4_, accompanied by optical opacity due to increased scattering on larger (~25–60 nm) silicate crystallites.

A detailed Rietveld refinement was performed on the set of selected samples (5Na-750-10, 5Na-760-10, 5Na-850-10, and others). The refinement employed structural models from the Crystallography Open Database (COD) [26] for ZnAl_2_O_4_ (ID 9007017, 9 reflections), Zn_2_SiO_4_ (ID 9014832, 98 reflections), and Mg_2_SiO_4_ (ID 9000535, 46 reflections). The lattice parameters (a), coherent scattering domain sizes (D), and metal site occupancies (Zn:Mg ratios) were refined simultaneously. Partial cation substitution between Zn^2+^ and Mg^2+^ sites was allowed, resulting in simulated intensities closely matching the experimental profiles. The flexible background “Chebyschev-1” function with 10 parameters was used to subtract diffuse peaks of glass phases.

**Figure 3:**
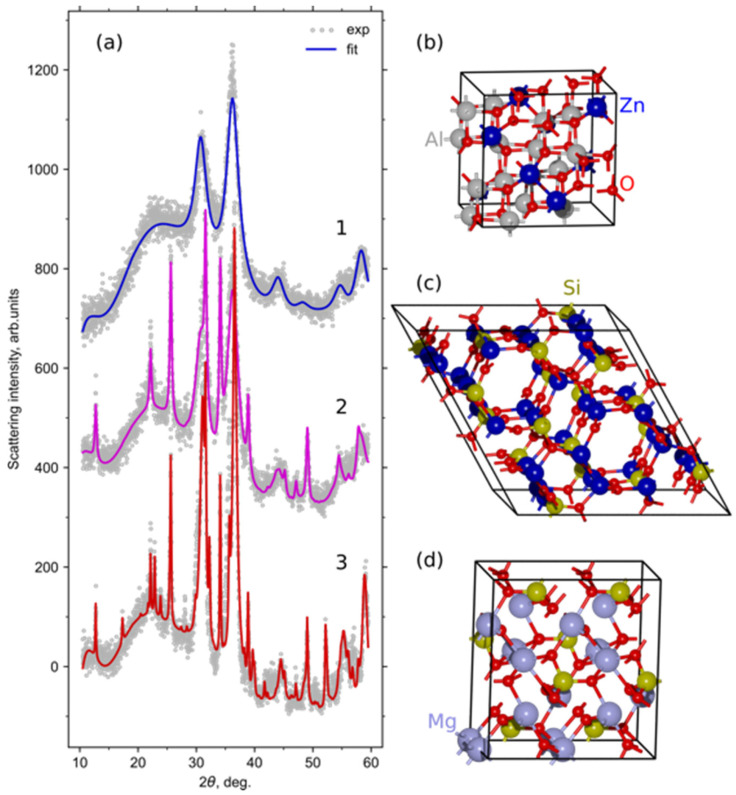
(**a**) Experimental XRD patterns (dots) and fitted theoretical curves (lines) obtained from Rietveld refinement for selected 5Na TGC: 1-5Na-750-10, 2-5Na-750-110, 3-5Na-850-10. Panels (**b**–**d**) illustrate the refined crystal structures of ZnAl_2_O_4_, Zn_2_SiO_4_, and Mg_2_SiO_4_ phases.

The experimental and calculated XRD patterns, along with corresponding fits, are shown in Figure 3, and the numerical results are summarized in Table 2. The refinement quality, expressed as the goodness of fit (GOF), ranged between 1.1 and 1.5, which is acceptable for nanocrystalline glass-ceramic systems with residual amorphous background. Representative fits for other samples are provided in the Supporting Information (Figures S3–S8).

A further improvement of the fitting quality may require variation in thermal vibration parameters, stresses, and size distributions influencing the XRD pattern. However, for the considered experimental data, such fine-tuning will give high cross-correlations between varied parameters and decrease the reliability of the obtained data. Also note that the interpretation of the obtained Zn:Mg ratios may be ambiguous since we cannot reliably distinguish the replacement of a heavier Zn atom (Z = 30) by a lighter Mg atom (Z = 12) and the formation of Zn vacancies by studying XRD data only. However, our interpretation is justified by the increase in electronic density in Mg silicate phases, which cannot be due to vacancy formations.

The refined parameters indicate that for transparent samples (e.g., 5Na-750-10 and 5Na-760-10), ZnAl_2_O_4_ is the only crystalline phase present. Its cubic lattice parameter is a = 8.21–8.23 Å, which is approximately 1% larger than that of the stoichiometric reference ZnAl_2_O_4_ (8.09 Å). This expansion likely results from partial substitution of Zn^2+^ by Mg^2+^ in tetrahedral sites, consistent with the refined cation occupancy ratios of Zn:Mg = 55:45–48:52.

With increasing temperature and holding time, the relative fraction of ZnAl_2_O_4_ decreases while silicate phases emerge. At 800 °C, the glass-ceramics consist of approximately 86 wt.% ZnAl_2_O_4_, 5 wt.% Zn_2_SiO_4_, and 9 wt.% Mg_2_SiO_4_, whereas at 850 °C, the phase composition shifts to 72 wt.% ZnAl_2_O_4_, 10 wt.% Zn_2_SiO_4_, and 18 wt.% Mg_2_SiO_4_. The silicate crystallites exhibit sizes between 25 and 60 nm, while spinel nanocrystals remain around 4–10 nm, preserving a fine-grained structure even at high temperatures.

No crystalline phases of ZrO_2_ or TiO_2_ were detected, which can be attributed to their low content in the base glass and the overlap of their most intense reflections with the broad spinel peaks. The refined results thus confirm that controlled crystallization at 750–760 °C for 10–25 h yields a uniform nanocrystalline ZnAl_2_O_4_ spinel phase with minor cation disorder (Zn–Mg intermixing), responsible for the combination of high transparency and mechanical stability.

**Figure 4:**
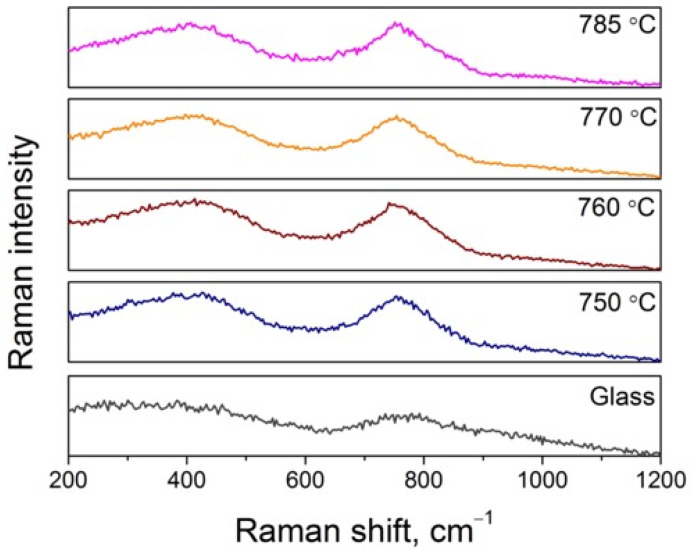
Raman spectra of the parent 5Na glass and TGCs heat-treated at different temperatures for 10 h.

Raman spectra of the parent glass and the glass-ceramics heat-treated at different temperatures are shown in Figure 4. The spectrum of the parent 5Na glass is broad and diffuse, exhibiting a weak, poorly resolved band near 800–850 cm^−1^, which can be attributed to Si–O and Al–O stretching vibrations in a chemically heterogeneous glass network. Such spectral broadening is typical of glasses undergoing liquid–liquid phase separation, which is also evident from the TEM observations showing nanometric contrast variations in the glass matrix.

Upon heat treatment at 750–785 °C, two distinct Raman bands become apparent at approximately 415 cm^−1^ and 760 cm^−1^. The former corresponds to Zn–O symmetric stretching vibrations in the cubic spinel ZnAl_2_O_4_ [27,28], while the latter is assigned to Al–O symmetric stretching vibrations of [AlO]_x_ units within the ZnAl_2_O_4_ lattice [29,30]. All spectra in this temperature range display the same set of features, and only the intensity of both bands increases slightly with temperature, indicating the growth and ordering of the gahnite nanocrystals rather than the formation of new phases. These observations confirm that the crystallization of the 5Na glass proceeds through phase-separation and the development of ZnAl_2_O_4_ nanocrystals, in full agreement with the XRD data.

**Figure 5:**
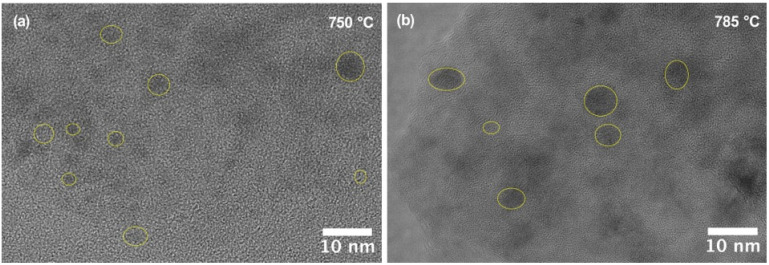
HRTEM images of TGC heat-treated at (**a**) 750 °C and (**b**) 785 °C. Yellow circles indicate ZnAl_2_O_4_ nanocrystals in the glassy matrix.

Representative HRTEM images of the glass-ceramics heat-treated at 750 °C and 785 °C are shown in Figure 5. After heat treatment at 750 °C (Figure 5a), the material exhibits a homogeneous nanostructure with uniformly dispersed crystalline domains of ZnAl_2_O_4_ embedded in the glassy matrix. The nanocrystals are nearly spherical and their average size does not exceed 4–5 nm, consistent with the value estimated from XRD data. Lattice fringes corresponding to the (311) and (400) planes of cubic ZnAl_2_O_4_ are locally resolved, confirming the single-phase spinel structure of the crystalline component.

When the crystallization temperature is increased to 785 °C (Figure 5b), the contrast variations become more pronounced, and individual nanocrystals grow slightly in size, reaching 6–8 nm. Nevertheless, the material still maintains a fine and uniformly distributed nanocrystalline structure without the formation of secondary crystalline phases or large aggregates. The observed microstructural evolution supports the Raman and XRD results, indicating that the heat treatment up to 785 °C promotes growth and ordering of gahnite nanocrystals.

### 3.3. Mechanical Properties and Ion-Exchange Strengthening

**Figure 6:**
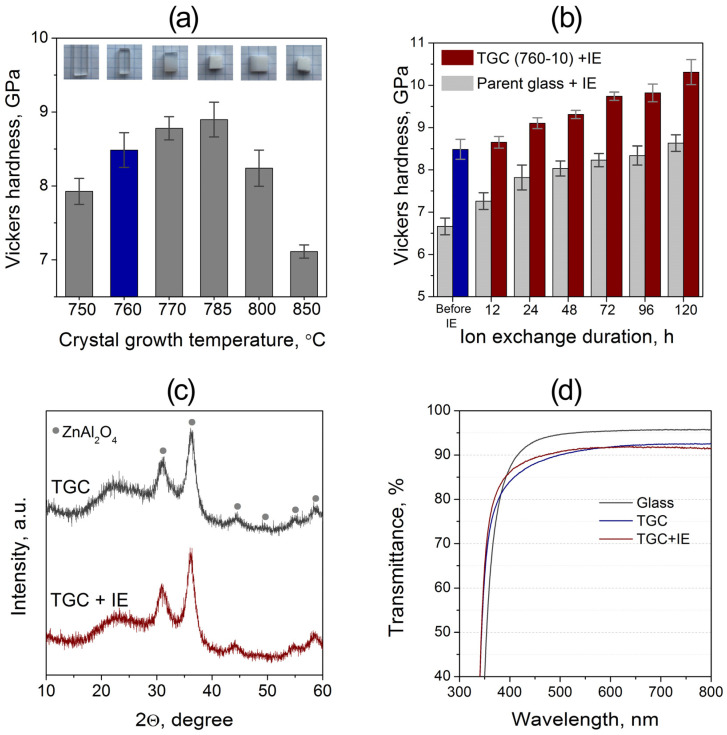
(**a**) Dependence of the Vickers hardness of 5Na glass-ceramics on the temperature of the crystal-growth stage (10 h). Insets show photographs of the samples illustrating the gradual loss of transparency with increasing crystallization temperature. (**b**) Evolution of Vickers hardness during IE for the parent glass and the TGC 5Na–760–10. (**c**) XRD patterns of the TGC before and after 120 h of IE. (**d**) Optical transmission spectra of the parent glass, the TGC, and the IE TGC.

The dependence of the Vickers hardness on the temperature of the crystal-growth stage is presented in Figure 6a. The parent 5Na glass shows an average hardness of 6.7 GPa. Two-stage heat treatment leads to a noticeable increase in hardness by about 1–2 GPa, yielding values in the range of 7.9–8.9 GPa depending on the temperature of the second stage. Samples treated at 750–760 °C remain fully transparent, while heating to 770 °C results in opalescence, and at 785 °C and above, the material becomes opaque. The highest hardness among the transparent glass-ceramics is achieved for the sample crystallized at 760 °C, reaching ≈8.5 GPa. The gradual rise in hardness up to 770–785 °C correlates with the growth and structural ordering of ZnAl_2_O_4_ nanocrystals revealed by TEM and Raman spectroscopy, whereas the subsequent decrease in hardness at higher temperatures is associated with the coarsening of crystallites and the appearance of secondary silicate phases, which deteriorate both the mechanical and optical properties.

The influence of IE in molten KNO_3_ at 450 °C on the hardness of the parent glass and of the TGC 5Na–760–10 is shown in Figure 6b. For both materials, the hardness increases steadily with the duration of IE, indicating the formation of a compressive stress layer as Na^+^ ions are replaced by K^+^ in the surface region. During the first 24 h, the increase in hardness is more pronounced for the glass, likely due to the absence of crystal–matrix interfaces that hinder alkali diffusion. At longer treatment times (≥ 24 h), the rate of hardness growth becomes similar for both materials. After 120 h, the hardness of the ion-exchanged TGC reaches ≈10–10.5 GPa, clearly exceeding that of the ion-exchanged glass. The higher strengthening efficiency in TGC arises from the restricted Na^+^ mobility in the nanocrystalline spinel matrix, which promotes a steeper concentration gradient and higher compressive stress near the surface. Although saturation is not yet reached after 120 h, further improvement would likely be marginal and of limited technological relevance given the long processing time required.

To determine whether prolonged contact with molten salt affects the crystalline structure of the TGC, XRD patterns of the 5Na–760–10 sample were recorded before and after 120 h of IE (Figure 6c). The diffraction profiles are identical and fully indexed to ZnAl_2_O_4_, showing no additional peaks or shifts, which indicates that the IE process does not modify the phase composition or lattice parameters of the crystalline phase. The optical transmission spectra (Figure 6d) confirm that the ion-exchanged TGC retains high transparency in the 400–800 nm range, with only a minor reduction compared to the parent glass.

**Figure 7:**
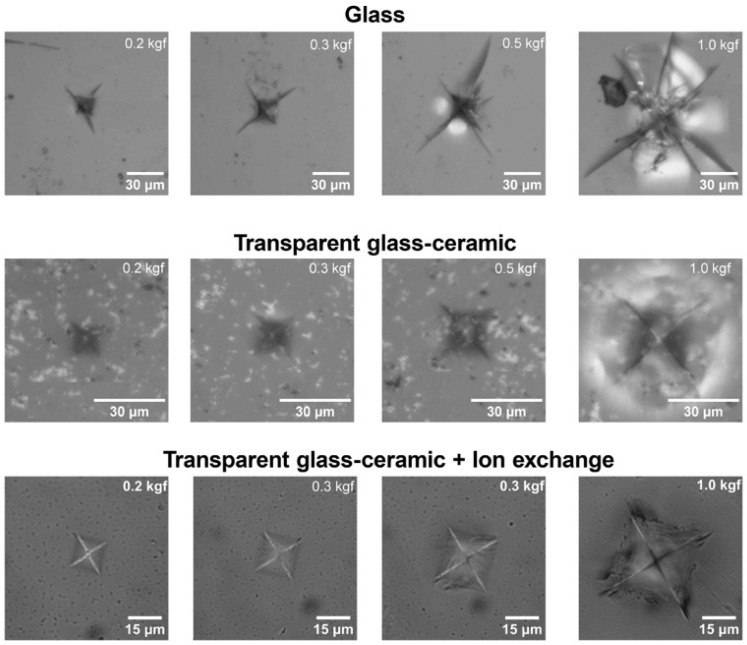
Optical micrographs of Vickers indents obtained under different loads (0.2–1.0 kgf) for (**top**) the parent glass, (**middle**) the TGC, and (**bottom**) the ion-exchanged TGC.

The optical micrographs of Vickers indents obtained under different loads for the parent glass, transparent glass-ceramic, and ion-exchanged glass-ceramic are shown in Figure 7. All imprints exhibit the characteristic square shape of the Vickers indenter. For the parent glass, radial cracks emanate from the corners of the indent even at the lowest applied load (0.2 kgf), and their number and length increase progressively with increasing load. After crystallization at 760 °C, the resulting TGC demonstrates a markedly improved crack resistance: no visible cracks are observed up to 0.5 kgf, and only at 1 kgf do minor damage zones appear around the indentation, associated with the brittle response of the residual glassy phase.

In contrast, the ion-exchanged TGC exhibits significantly enhanced resistance to indentation-induced fracture. Even at the maximum applied load (1 kgf), only small, short cracks appear from some indent corners, while the surrounding surface remains intact and free from mirror-like reflections typical of brittle fracture. This behavior confirms that the combination of a fine-grained ZnAl_2_O_4_ nanocrystalline framework and the compressive surface layer formed during ion exchange effectively suppresses crack propagation and improves the overall damage tolerance of the material.

Together with the hardness data, these results demonstrate that moderate crystallization at 750–760 °C followed by ion-exchange treatment in molten KNO_3_ yields transparent glass-ceramics possessing both high surface hardness (>10 GPa) and outstanding resistance to cracking—properties crucial for mechanically loaded transparent components.

The obtained results can be compared with previously reported data summarized in Table 1. For most alkali-containing transparent glass-ceramics, the microhardness before ion exchange typically lies in the range of 6–8 GPa, increasing to 7–9 GPa after IE treatment [9,10,11,12,13,14,15,16,18,20]. In contrast, the present ZnO–MgO–Al_2_O_3_–SiO_2_ system demonstrates higher hardness levels (8.5 GPa before IE and ≈10–10.5 GPa after IE) while maintaining high optical transparency in the 400–800 nm range. This performance surpasses that of previously studied ZMAS-type glass-ceramics [13,14], where coarse or mixed-phase crystallization limited transparency and further ion-exchange efficiency. The superior mechanical response of the present material can be attributed to the formation of a uniform nanocrystalline ZnAl_2_O_4_ spinel network combined with a compressive surface layer produced during ion exchange, which together ensure both high hardness and resistance to crack propagation.

## 4. Conclusions

Transparent ZMAS glass-ceramics containing 5 mol% Na_2_O were successfully fabricated via a controlled two-stage heat treatment. The parent glass exhibited an amorphous structure with nanoscale compositional fluctuations, indicating the occurrence of liquid–liquid phase separation. DSC and XRD analyses revealed two exothermic crystallization events, while controlled crystallization at 660 °C for 4 h and 750–760 °C for 10 h produced transparent glass-ceramics consisting solely of ZnAl_2_O_4_ (gahnite) nanocrystals with an average size of 4–5 nm. Raman spectroscopy and HRTEM confirmed that increasing the temperature to 785 °C leads only to the growth and ordering of the spinel phase, without the formation of secondary silicates.

The optimal combination of high transparency and mechanical strength was achieved for samples crystallized at 760 °C, exhibiting a Vickers hardness of ≈8.5 GPa. Subsequent ion exchange in molten KNO_3_ at 450 °C further increased the hardness to ≈10–10.5 GPa without altering the phase composition or transparency. The strengthened samples demonstrated a pronounced resistance to crack initiation and propagation under indentation.

Compared with literature data on alkali-containing transparent glass-ceramics (Table 1), the obtained material shows a higher hardness level while maintaining optical transparency, indicating the efficiency of the selected composition and processing route. Overall, the study demonstrates that moderate crystallization followed by ion exchange provides an effective route to produce transparent, mechanically durable ZnAl_2_O_4_-based glass-ceramics, combining optical clarity with enhanced surface hardness. Such materials are promising for applications requiring high optical transparency and mechanical reliability, including protective and structural components in optical and photonic devices, display and cover glasses, micro-optical elements, and transparent armor systems, where improved resistance to surface damage and cracking is critical. Future work will focus on evaluating the thermal-shock resistance, long-term stability, and scalability of the synthesis and ion-exchange strengthening processes to assess their potential for practical implementation.

## Figures and Tables

**Table 1:** Literature data on the composition and properties of TGC before and after ion-exchange strengthening.

Glass System and Alkali Oxide Content (mol.%)	Main Crystalline Phase	Salt Composition and IE Treatment Conditions	Vickers Hardness, GPa (Load, kgf)	Ref.
LAS Li_2_O—21 Na_2_O—1	Li_2_Si_2_O_5_ LiAlSi_4_O_10_	Before IE Na_0.95_K_0.05_NO_3_ 410 °С—4 h 410 °С—6 h 410 °С—8 h 410 °С—10 h	7.78 (n/a) 8.53 (n/a) 8.87 (n/a) 8.64 (n/a) 8.55 (n/a)	[9]
LAS Li_2_O—15 Na_2_O—9 K_2_O—1	LiAlSiO_4_	Before IE (Na_0.8_K_0.2_)NO_3_ 420 °C—4 h	7.03 (0.2) 7.62 (0.2)	[10]
LAS Li_2_O—21.6	LiAlSi_4_O_10_	Before IE NaNO_3_ 450 °C—6 h	6.95 (0.2) 7.45 (0.2)	[11]
LS Li_2_O—27.5	Li_2_Si_2_O_5_	Before IE NaNO_3_ 315 °C—12 h 385 °С—1 h 450 °С—1 h	5.9 (0.05) 6.5 (0.05) 6.3 (0.05) 6.2 (0.05)	[12]
ZMAS Na_2_O—8	ZnAl_2_O_4_ MgAl_2_O_4_	Before IE KNO_3_ 440 °C—4 h	7.2 (0.2) 8.3 (0.2)	[13]
ZAS Na_2_O—8.5	Zn_2_TiO_4_ Zn_2_SiO_4_	Before IE KNO_3_ 410 °C—4 h 430 °С—4 h 450 °С—4 h	6.6 (0.2) 7.8 (0.2) 7.7 (0.2) 7.6 (0.2)	[14]
LZAS Li_2_O—5.95	ZnAl_2_O_4_ LiAlSi_4_O_10_	Before IE NaNO_3_ 460 °C—4 h -> KNO_3_ 460 °C—4 h	7.45 (0.2) 8.1 (0.2)	[15]
ZAS Na_2_O–10.5	ZnO Zn_2_SiO_4_	Before IE KNO_3_ 460 °C—8 h	6.3 (0.2) 7.6 (0.2)	[16]
LAS Li_2_O—12.4 Na_2_O—2.8	β-SiO_2_ LiAlSi_2_O_6_ Li_2_SiO_3_	Before IE KNO_3_ 400 °C—10 h 420 °C—10 h 440 °C—10 h	5.2 (0.01) 7.3 (0.01) 7.4 (0.01) 7.7 (0.01)	[18]
NAS Na_2_O—15	NaAlSiO_4_	Before IE KNO_3_ 600 °C—8 h	4.4 (n/d) 6.3(n/d)	[20]
LAS Li_2_O—15 Na_2_O—9	LiAlSiO_4_ NaAlSiO_4_	Before IE Na_0.85_K_0.15_NO_3_ 430 °C—4 h 450 °C—4 h 470 °C—4 h 490 °C—4 h -> KNO_3_ 380 °C—4 h 400 °C—4 h 420 °C—4 h 440 °C—4 h	7.4 (0.2) 7.5 (0.2) 7.5 (0.2) 7.5 (0.2) 7.5 (0.2) 8.0 (0.2) 8.1 (0.2) 8.2 (0.2) 8.1 (0.2)	[21]
ZMAS Na_2_O—5.9	MgAl_2_O_4_ ZnAl_2_O_4_ SnO_2_ ZrO_2_	Before IE Na_0.02_K_0.98_NO_3_ 450 °C—5 h 470 °C—5 h 490 °C—5 h	7.0 (0.2) 8.0 (0.2) 8.1 (0.2) 8.2 (0.2)	[22]

**Table 2:** Refined structural parameters of crystalline phases in 5Na TGC obtained from Rietveld analysis: lattice parameters (a), crystallite size (D), cation occupancies (Zn:Mg), phase fractions, and goodness of fit (GOF). The measurement uncertainty falling at the last digit is shown in brackets.

Sample	Crystalline Phase	Phase Fraction, wt.%	Cell Parameters, Å	Crystallite Size, nm	Site Occupancy Zn:Mg	GOF
5Na-750-10	ZnAl_2_O_4_	100	8.223 (3)	3.7 (1)	55:45 (2)	1.52
5Na-760-10	ZnAl_2_O_4_	100	8.207 (3)	4.1 (1)	52:48 (2)	1.27

5Na-750-25	ZnAl_2_O_4_	100	8.234 (3)	3.8 (1)	48:52 (2)	1.27

5Na-750-110	ZnAl_2_O_4_	83	8.220 (3)	3.9 (1)	57:43 (2)	1.35
	Zn_2_SiO_4_	17	13.905 (4), 9.302 (2)	25.8 (7)	45:55 (5)	

5Na-800-10	ZnAl_2_O_4_	86	8.205 (2)	5.0 (1)	62:38 (1)	1.39
	Zn_2_SiO_4_	5	13.94 (2), 9.28 (1)	12 (1)	100:0 *	
	Mg_2_SiO_4_	9	4.762 (4), 10.222 (7), 6.000 (5)	28.8 (4)	0:100 *	

5Na-850-10	ZnAl_2_O_4_	72	8.141 (2)	9.9 (2)	81:19 (3)	2.07
	Zn_2_SiO_4_	10	13.909 (4), 9.307 (2)	66 (5)	80:20 (10)	
	Mg_2_SiO_4_	18	4.769 (2), 10.239 (4), 5.982 (3)	56 (6)	15:85 (3)	

5Na-700-24	ZnAl_2_O_4_	80	8.204 (6)	3.3 (1)	49:51 (3)	1.12
	Zn_2_SiO_4_	7	14.51 (3), 8.38(1)	3 (1)	100:0 *	
	Mg_2_SiO_4_	13	4.69 (3), 10.09 (6), 6.08 (4)	7 (2)	0:100 *	

Reference	ZnAl_2_O_4_	-	8.085	-	-	-
	Zn_2_SiO_4_	-	13.971, 9.334	-	-	
	Mg_2_SiO_4_	-	4.752, 10.193, 5.977	-	-	

* Site occupancies for minor phases were fixed due to their small fraction.

## Data Availability

The original contributions presented in this study are included in the article/Supplementary Material. Further inquiries can be directed to the corresponding author.

## Supplementary Materials

The following supporting information can be downloaded at: https://www.mdpi.com/article/10.3390/ma18225230/s1, Figure S1: DSC measurements on glass samples of identical mass in powdered and monolithic forms; Figure S2: XRD patterns for powdered and monolithic samples heat-treated under identical conditions 660 °C—4 h—760 °C—10 h; Figures S3–S8: The illustration of fitting qualities of the XRD patterns for the samples.
