# Supplementary material for: Ultra-Strong Transparent ZnAl2O4 Glass-Ceramics via Controlled Crystallization and Ion Exchange
Source: Materials (Basel). 2025 Nov 19;18(22):5230. doi: 10.3390/ma18225230 (PMC12654701; doi:10.3390/ma18225230)
Supplement: Supplementary file 1 [file materials-18-05230-s001.zip › materials-3985550-supplementary.pdf]

Supplementary material

Ultra-Strong Transparent $ZnAl_2O_4$ Glass-Ceramics via Controlled Crystallization and Ion Exchange

Ivan Veselov1, Georgiy Shakhgildyan1*, Vitaliy Savinkov1, Nikita Golubev1, Kirill Tregubov1, Daniil Vinogradov1, Leon Avakyan2, Michael Ojovan3, Manasi Ghosh4, Vladimir Sigaev1

[1] Mendeleev University of Chemical Technology, Moscow, Russia, shakhgildian.g.i@muctr.ru
[2] Southern Federal University, Rostov-on-Don, Russia
[3] School of Chemical, Materials and Biological Engineering, The University of Sheffield, Sheffield S1 3JD, UK
[4] Physics Section, MMV, Banaras Hindu University, Varanasi 221005, India
* Correspondence: shakhgildian.g.i@muctr.ru

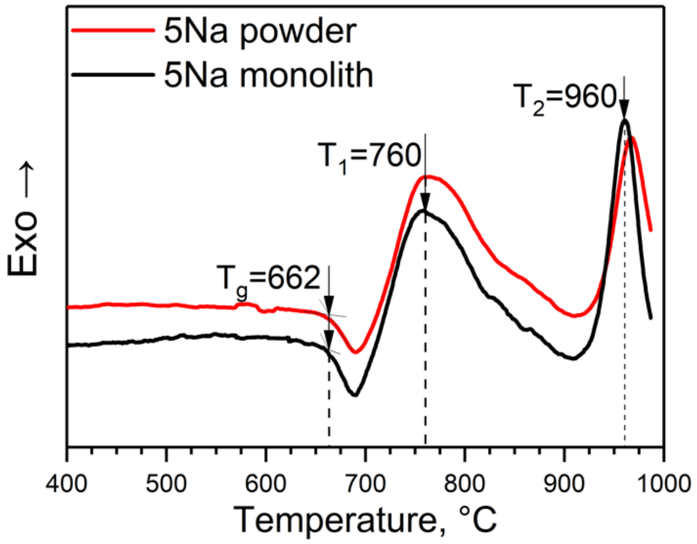

Figure S1. DSC measurements on glass samples of identical mass in powdered and monolithic forms.

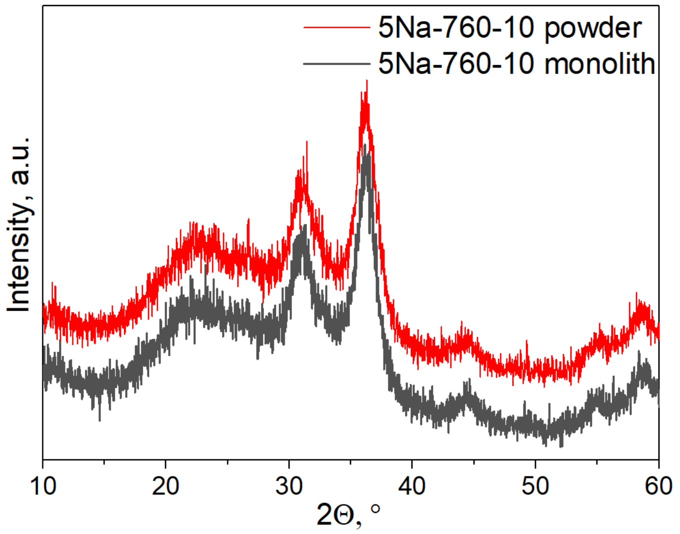

Figure S2. XRD patterns for powdered and monolithic samples heat-treated under identical conditions 660 °C – 4 h – 760 °C – 10 h.

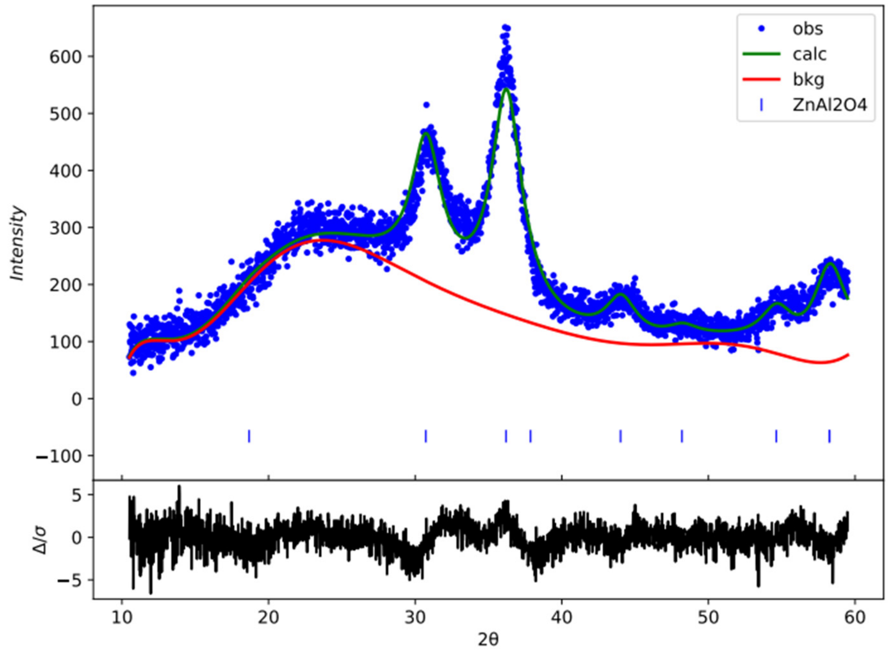

Figure S3. The illustration of fitting qualities of the XRD patterns for the sample 5Na4-660-4-750-10

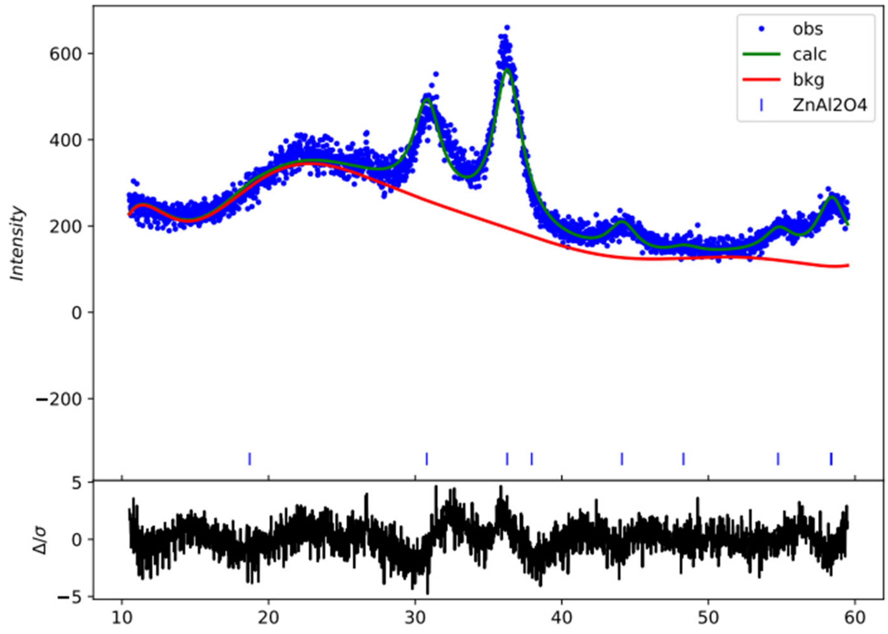

Figure S4. The illustration of fitting qualities of the XRD patterns for the sample 5Na4-660-4-760-10

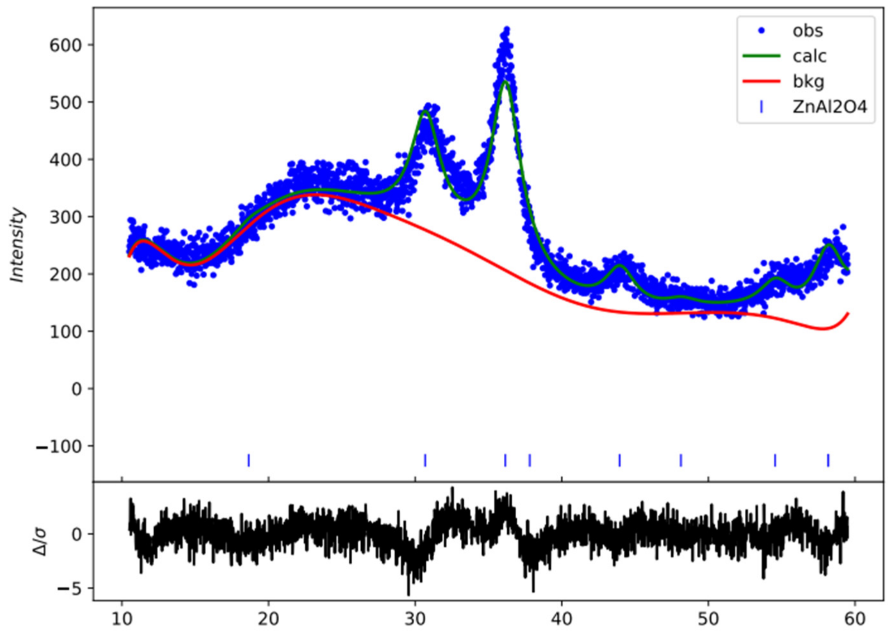

Figure S5. The illustration of fitting qualities of the XRD patterns for the sample 5Na4-660-4-750-25

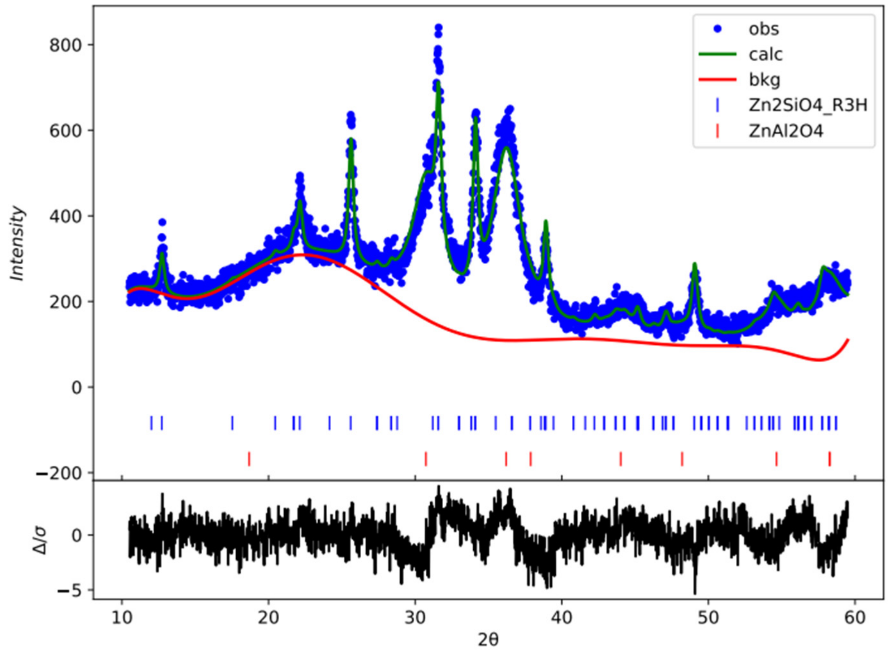

Figure S6. The illustration of fitting qualities of the XRD patterns for the sample 5Na4-660-4-750-110

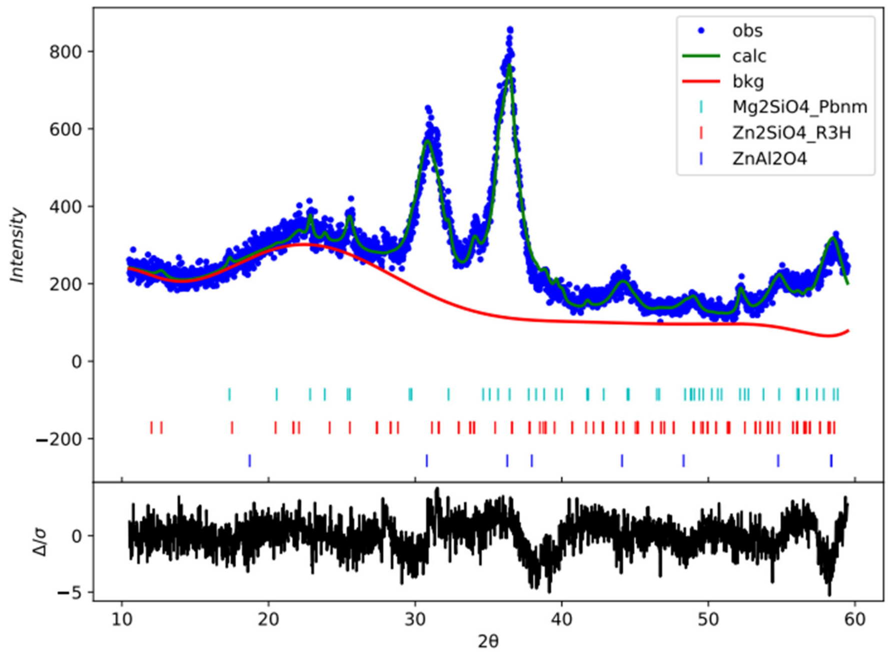

Figure S7. The illustration of fitting qualities of the XRD patterns for the sample 5Na4-660-4-800-10

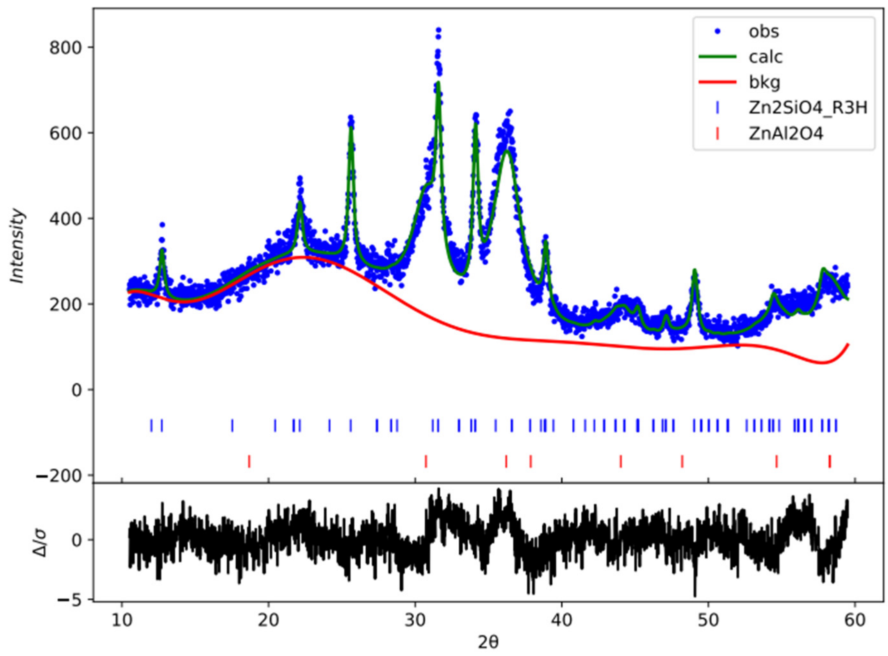

Figure S8. The illustration of fitting qualities of the XRD patterns for the sample 5Na4-660-4-850-10
